# Prevalence and patterns of biochemical discordance in patients with surgically treated acromegaly

**DOI:** 10.1007/s11102-026-01694-6

**Published:** 2026-05-23

**Authors:** Anna Pennlund, Thomas Olsson Bontell, Thomas Skoglund, Helena Carén, Gudmundur Johannsson, Daniel S. Olsson, Daniela Esposito

**Affiliations:** 1 Department of Clinical Pathology, Sahlgrenska University Hospital, Gula Stråket 8, Gothenburg, 413 45 Sweden; 2 Department of Internal Medicine and Clinical Nutrition, Institute of Medicine, Sahlgrenska Academy, University of Gothenburg, Gothenburg, Sweden; 3 Department of Physiology, Institute of Neuroscience and Physiology, Sahlgrenska Academy, University of Gothenburg, Gothenburg, Sweden; 4 Department of Neurosurgery, Sahlgrenska University Hospital, Gothenburg, Sweden; 5 Department of Clinical Neuroscience, Institute of Neuroscience and Physiology, Sahlgrenska Academy, University of Gothenburg, Gothenburg, Sweden; 6 Sahlgrenska Center for Cancer Research, Department of Medical Biochemistry and Cell Biology, Institute of Biomedicine, Sahlgrenska Academy, University of Gothenburg, Gothenburg, Sweden; 7 Department of Endocrinology, Sahlgrenska University Hospital, Gothenburg, Sweden

**Keywords:** Acromegaly, Biochemical discordance, Growth hormone, Insulin-like growth factor-1, Pituitary adenoma, Pituitary neuroendocrine tumour

## Abstract

**Objective:**

Discordance between growth hormone (GH) and insulin-like growth factor-1 (IGF-1), where one hormone is within the age- and sex-adjusted reference and the other is not, can be observed in patients with acromegaly. The discrepancy complicates the interpretation of disease activity, thereby presenting a significant challenge in the clinical management of acromegaly. This study aimed to assess postoperative discordance prevalence and clinical correlates to clarify the implication of discordance.

**Methods:**

This retrospective study describes a cohort of patients that has undergone pituitary surgery at Sahlgrenska University Hospital between 1994 and 2019 due to acromegaly. Medical records were reviewed and the group with postoperative discordant hormones was compared with the group with concordant hormones.

**Results:**

In a cohort of 82 patients surgically treated for acromegaly and thereafter examined with an oral glucose tolerance test (OGTT), 16 (19.5%) exhibited biochemical discordance. Fourteen of 16 patients showed elevated IGF-1 but normal GH. The IGF-1 levels at diagnosis were significantly higher in patients with discordance compared with the controlled concordant group. However, no differences were observed between the discordant and the concordant group regarding the baseline variables at diagnosis: age, gender, invasive tumour, micro/macro-adenoma or BMI, nor hypertensive treatment at time of surgery and postoperative radiotherapy or reoperation.

**Conclusion:**

The prevalence of biochemical discordance in our cohort was 19.5%. The predominant pattern was elevated IGF-1 levels with a normal nadir GH. The discordant group had higher IGF-1 at diagnosis, suggesting that greater preoperative disease activity may predispose to postoperative biochemical discordance.

## Introduction

Acromegaly primarily results from chronic overproduction of growth hormone (GH) from a pituitary neuroendocrine tumour (pitNET) also known as a pituitary adenoma [1, 2]. Laboratory findings in patients with surgically treated acromegaly often show biochemical concordance between GH and insulin-like growth factor-1 (IGF-1) levels, meaning either both indicate biochemical remission or not. Some patients may however exhibit discordant values of these hormones, referred to as biochemical discordance. Previous studies report discordance frequencies of 19.2–35% [3–6] and a review article from 2016, estimated the pooled frequency to 25.7%, where elevated IGF-1 and normal GH value was the most common type of discordance [7].

Discordance in acromegaly has been attributed to several factors [8] since multiple variables can impact GH and IGF-1 levels: analytical challenges in hormone measurements [9, 10]; kidney failure [11]; liver disease [12]; oestrogen replacement therapy [13]; treatment with GH receptor antagonist [14] or somatostatin receptor ligand (SRL) [7]. Reports also suggest that body mass index (BMI) may affect the risk of discordant values [15] and that the rate and type of discordance is affected by the used cut-off for GH level [16]. In one study [17], spontaneous normalisation of serum IGF-1 up to 57 months after surgery occurred in patients with discordant values (and no residual tumour).

The aim of this study was to examine the prevalence of postoperative discordance in a large cohort of patients with acromegaly treated with pituitary surgery at a single centre. The secondary aim was to study clinical patterns of the group of patients with discordant GH and IGF-1 in comparison with those exhibiting concordant values, to better understand the implication of discordance.

## Materials and methods

### Data gathering

This retrospective single-centre study includes patients with acromegaly that have undergone pituitary surgery due to a pitNET causing acromegaly, at Sahlgrenska University Hospital between year 1994–2019. Data were collected from three separate record systems up to the end of the study period (November 2022); the booking system used at the Department of Neurosurgery, the general medical records used at the Department of Endocrinology and the record system of the Department of Clinical Pathology. Follow-up data were collected at standardised intervals from the date of surgery (allowable window in parentheses): 3 months (1–7 months post-surgery date); 1 year (± 3 months); 2 years (± 6 months); 5 years (± 9 months); and 10 years (± 12 months). The selection process, assessment of biochemical control, tumour invasiveness and size on magnetic resonance imaging (MRI) are described in detail in our earlier study, on which this cohort is based [18]. To allow for comparison between patients’ IGF-1 samples, the age- and sex-adjusted IGF-1 ratio of the upper limit of normal (ULN) was used in the analysis. For this current study, additional inclusion criteria were applied: The patients were required to have performed at least one oral glucose tolerance test (OGTT) during the period between 3-months and 2-years postoperative follow-up (actual time frame 1 month–2.5 years post-surgery) and not received treatment with a SRL or a GH receptor antagonist within one month from the displayed discordant values. Patients exhibiting biochemical discordance—defined as the presence of one parameter (GH or IGF-1) within the reference range for remission while the other remained elevated—were categorised as the “discordant group”. A subgroup of patients that had not displayed biochemical discordance were examined with regard to disease control at the 2-year (± 6 months) postoperative follow-up.

### Data analysis

Between 1994 and 2022, multiple assays have been used for analysis of serum GH and IGF-1. In this cohort, the assays used for IGF-1 were Nichols RIA (1994–2003), Nichols Advantage (2003–2006), Siemens Immulite 2500 (2006–2011), Siemens Immulite 2000XPi (2011–2013) and IDS-iSYS IGF1 IS-3900 (2013–2022). The assays used to measure GH were: hGH RIA kit 10-6409-01 (1994–1995), DELFIA hGH kit nr 1244.041 (1995–2008), and Reagent Access GH, ref 33,580 (2008–2022). In this study, the cut-off values used for assessment of GH during an OGTT was set to 1.0 µg/L before 2010 and 0.4 µg/L after, based on two papers of consensus criteria [19, 20]. For samples where unit conversion from mU/L to µg/L was required, the formula based on a study for standardisation of GH assays was used [21].

BMI was calculated ($\:kg/{m}^{2}$) and categorised according to World Health Organization criteria [22].

Statistical analyses were performed in IBM SPSS Statistics 25. A p-value of < 0.05 was set as a statistically significant result. For comparing the discordant and the concordant group, Chi-Squared Test and Fisher’s Exact Test was used for categorical variables and Mann–Whitney U Test for continuous variables.

## Results

**Fig. 1:**
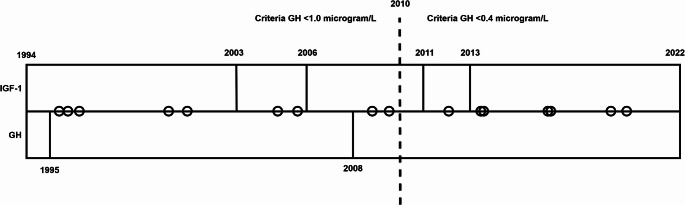
Graph depicting a timeline of used assays and discordant values. The graph shows two swim lanes representing the different GH and IGF-1 assays used between 1994–2022. Circles represent a patient and indicate the time of the first post-surgical discordant value. The dotted line represents the time point of change in the GH cut-off value during OGTT from 1.0 to 0.4 µg/L

A cohort of 103 patients (60 men, 43 women) were treated with pituitary surgery and diagnosed with a GH- or GH/prolactin-secreting pitNET at Sahlgrenska University Hospital between year 1994–2019, of whom 82 patients met the study inclusion criteria (Table 1). A total of 20 individuals were excluded due to missing IGF-1 and a GH during OGTT within the predefined time frame and one individual due to treatment with SRL within one month of the displayed discordant value. Of the 82 individuals, 16 (19.5%) exhibited discordant GH and IGF-1 values and were classified as the “discordant group” (12 men, 4 women) with a median age of 43 (range:30–75 years). Concordant values were recorded in the remaining 66 (80.5%) patients (36 men, 30 women) with a median age of 47 (range:23–74 years, missing data *n* = 1). The most common type of discordant constellation was an elevated IGF-1 and GH within reference for biochemical control of acromegaly (14/16, 87.5%). The time of the first exhibited discordant sample for each individual is depicted in Fig. 1. The cases were distributed across the study period and various assays, with no discernible temporal clustering or assay-specific pattern. The group was too small to determine whether specific GH and IGF-1 assays predisposed patients to biochemical discordance.

**Table 1:** Discordant and concordant cases and their characteristics

Parameter	Discordant, *n* = 16 (%)	Concordant, *n* = 66 (%)
Age at diagnosis, median (range)	43 (30–75)	47 (23–74) n/a, *n =* 1
Gender		
Men	12 (75)	36 (54.5)
Women	4 (25)	30 (45.5)
Discordance type		
High IGF-1/Normal GH	14 (87.5)	–
Normal IGF-1/High GH	2 (12.5)	–
Tumour residue post-op MRI	n/a, *n* = 6	n/a, *n* = 15
Yes	4 (40)	24 (47.1)
No	6 (60)	27 (52.9)
Tumour type		
GH	9 (56.3)	33 (50)
GH/PRL	7 (43.8)	33 (50)
Tumour size	n/a, *n* = 1	n/a, *n* = 1
Macroadenoma	13 (86.7)	48 (73.8)
Microadenoma	2 (13.3)	17 (26.2)
Invasive tumour	n/a, *n* = 1	n/a, *n* = 3
Yes	7 (46.7)	26 (41.3)
No	8 (53.3)	37 (58.7)
Diabetes treatment		n/a, *n* = 1
Yes	3 (18.8)	11 (16.9)
No	13 (81.3)	54 (83.1)
Hypertensive treatment		n/a, *n* = 2
Yes	6 (37.5)	22 (34.4)
No	10 (62.5)	42 (65.6)
BMI weight class	n/a, *n* = 3	n/a, *n* = 6
Normal (18.5–24.9)	3 (23.1)	18 (30)
Overweight (25–29.9)	5 (38.5)	30 (50)
Obesity class 1 (30–34.9)	4 (30.8)	10 (16.7)
Obesity class 2 (35–39.9)	1 (7.7)	1 (1.7)
Obesity class 3 (≥ 40)	0 (0)	1 (1.7)

(*N/a* not applicable)

## Analysis of the discordant group

In the group of 16 patients with a post-surgical discordant value, there were 12 men and 4 women. In 10 patients IGF-1 and nadir GH were measured in more than one occasion, with six patients showing discrepant values more than once (four patients had discrepant values over different IGF-1 assays). Of those who exhibited discrepant hormones only in one occasion, two patients had their follow-up samples taken earlier than three months from the surgery date (both at two months after surgery), one of them was lost to follow-up and the second patient exhibited uncontrolled acromegaly with concordant values at the 5-year postsurgical follow-up.

Eight patients underwent 5-year postoperative follow-up visit but only three with a new OGTT, where two patients exhibited discrepancy and one was concordant uncontrolled. The other patients were only monitored with IGF-1 at that point and their IGF-1 values ranged between 0.48–1.17×ULN. Concerning treatment for hypertension and diabetes as the 5-year postoperative follow-up; 4 out of 8 patients had treatment for hypertension but none was medicated for diabetes mellitus. Only one patient switched discordance type, from having had a slightly elevated IGF-1 (1.1×ULN) but normal GH at the 1-year postoperative follow-up to an elevated GH but normal IGF-1 at the 10-year follow-up.

At the time of surgery, six patients (38%, 6/16) had treatment for hypertension and three patients (19%, 3/16) had treatment for diabetes mellitus.

No patient was receiving oestrogen replacement therapy, GH receptor antagonist treatment or had a known liver disease at the time of the discordant results. Two patients had received thyroxin replacement, and two other patients received testosterone replacement; one of the latter had also undergone a nephrectomy due to previous kidney cancer (normal renal function at time of pituitary surgery, five years after nephrectomy). A total of 13/15 patients (missing data *n* = 1) were diagnosed with a macroadenoma and four individuals had a reported residual tumour on the postoperative MRI scan. In the subgroup of patients with residual tumour, three had a macroadenoma at diagnosis and the fourth a microadenoma (the latter was one of the patients with postoperative elevated GH and IGF-1 within reference).

**Fig. 2:**
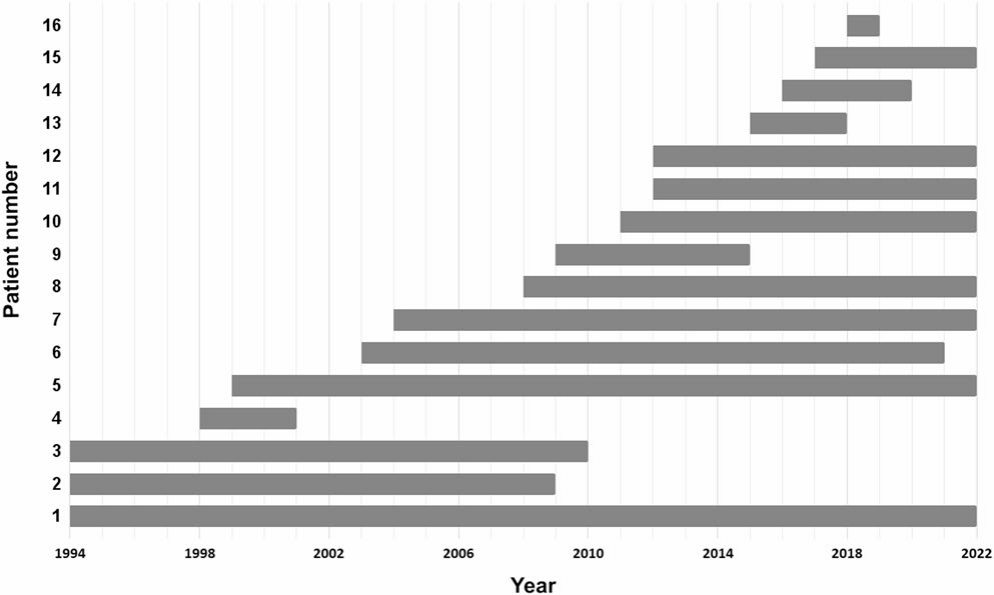
Overview of the follow-up duration from the time of diagnosis, of the subgroup with discordant values

The duration of follow-up varied between 1 and 28 years. An overview of the individual patients’ follow-up is presented in Fig. 2. An OGTT was not performed at every follow-up (3-months, 1-, 2- 5- and 10-years postoperative follow-up), mainly IGF-1 was sampled. During the individuals’ follow-up periods, 7 of 16 (43.8%) patients in the discordant group had at least one follow-up with either a combination of GH at an OGTT and IGF-1 or only an IGF-1 within the diagnostic criteria of biochemical control, whereas the concordant group displayed 55 of 66 (83.3%) of patients reaching biochemical control. Two of 16 (12.5%) patients in the discordant group received medical treatment with SRLs during their follow-up period. Specifically, the patients receiving SRLs, were those exhibiting elevated GH and an IGF-1 within age- and sex-adjusted reference range. One additional patient with high IGF-1 was treated with radiation therapy, whereas the remaining patients did not receive any treatment for acromegaly.

Comparison between the discordant and the concordant group showed no difference in the following variables at diagnosis: age, gender, IGF-1, invasive tumour, micro/macro-adenoma or BMI, nor hypertensive treatment at time of surgery (Table 2). No difference in the frequency of postoperative radiotherapy or reoperation was detected between the groups.

**Table 2:** Statistical analyses of the discordant and the concordant group. (N/a, not applicable.)

Parameter		Discordant, *n* = 16	Concordant, *n* = 66	*p*-value
Patient characteristic				
Age at diagnosis, median (range) (n/a)	43 (30–75)		47 (23–74) (1)	0.32
Gender, men/women	12/4		36/30	0.14
IGF-1 at diagnosis, mean (± SD) (n/a)	3.4 (± 1) (1)		2.9 (± 1.2) (3)	0.59
BMI, median (range), (na)	26.9 (24–33.6) (3)		26.8 (18.9– 40.1) (6)	0.37
Hypertensive treatment, yes/no (n/a)	6/10		22/42 (2)	0.82
Tumour characteristic				
Diameter mm, median (range) (n/a)	15 (4–22) (1)		15 (3–60) (4)	0.9
Adenoma, macro/micro (n/a)	13/2 (1)		48/17 (1)	0.50
Invasiveness, yes/no (n/a)	7/8 (1)		26/37 (3)	0.70
Reoperation, yes/no	0/16		4/62	0.58
Radiation, yes/no	1/15		2/64	0.48

(*N/a* not applicable)

### Analysis of discordant, controlled and uncontrolled patients

At the 2-year postoperative follow-up all of the patients from the discordant group (*n* = 16) had available follow-up data. In the patients with concordant GH and IGF-1, 53 had a recorded follow-up. Of those, 37 patients (70%) had controlled acromegaly and 16 (30%) uncontrolled disease. In Table 3, the discordant group is compared to the concordant group split into subgroups of controlled and uncontrolled disease with regard to baseline data at diagnosis: age, gender, IGF-1, invasive tumour, micro/macro-adenoma and BMI, hypertensive treatment at time of surgery, having received postoperative radiotherapy or reoperation. No differences were observed between the groups for any variables except for IGF-1 levels at diagnosis. Specifically, in the discordant group, median IGF-1 at diagnosis was significantly higher compared with the controlled concordant group (3.1 versus 2.5, *p* = 0.01).

**Table 3:** Analysis of baseline data of the discordant versus controlled concordant group (p-value 1) and discordant versus uncontrolled concordant group at the 2-year postoperative follow-up (p-value 2)

Parameter	Discordant, *n* = 16	Controlled, *n* = 37	*p*-value 1	Uncontrolled, *n* = 16	*p*-value 2
Patient characteristic					
Age at diagnosis, median (range) (n/a)	43 (30–75)	47 (23–69) (1)	0.23	37.5 (25–67)	0.41
Gender, men/women	12/4	18/19	0.13	10/6	0.70
IGF-1 at diagnosis, median (range) (n/a)	3.1 (1.8–5.8) (1)	2.5 (1–5.3) (2)	0.01	3.3 (1.7–6) (1)	0.25
BMI, median (range) (n/a)	26.9 (24–33.6) (3)	26.8 (18.9–40.9) (3)	0.23	27.8 (20.7–33.6) (1)	0.82
Hypertensive treatment, yes/no (n/a)	6/10	14/23	1.00	4/11 (1)	0.70
Tumour characteristic					
Diameter mm, median (range) (n/a)	15 (4–22) (1)	13.5 (3–60) (3)	0.49	23 (6–52) (1)	0.12
Adenoma, macro/micro (n/a)	13/2 (1)	25/11 (1)	0.30	14/2	1.00
Invasiveness, yes/no (n/a)	7/8 (1)	8/27 (2)	0.10	11/4 (1)	0.26
Reoperation, yes/no	0/16	1/36	1.00	3/13	0.23
Radiation, yes/no	1/15	0/37	0.30	2/14	1.00

(*N/a* not applicable)

Statistical comparison was made between the subgroup of discordant patients with elevated IGF-1 and GH below the diagnostic criteria of acromegaly during an OGTT (*n* = 14), versus the controlled concordant and uncontrolled concordant group with regard to the same variables used in the main analysis including all the discrepant cases. The result shows again, a statistical difference only between median IGF-1 at diagnosis when the discordant and the controlled concordant group is compared (median IGF-1 larger in the discordant group at 3.2 vs. concordant controlled 2.5, *p* = 0.002).

## Discussion

In our large single-centre cohort of patients surgically treated for acromegaly, the prevalence of discordance between GH and IGF-1 was approximately 20%. Most patients with discordance were men (75%) and exhibited an elevated IGF-1 but GH within reference of the diagnostic criteria for biochemical control of acromegaly (88%) applied at the time period.

Several physiological, pathological, and methodological factors may have an impact on GH and IGF-1 levels in patients with acromegaly, potentially affecting the rate of biochemical discordance. Elevated IGF-1 with normal GH is generally the more frequently reported pattern of biochemical discordance, with a recent systematic review and meta-analysis showing a frequency of 15% in a total of 7071 patients from 39 studies [7]. In agreement with these findings, 17% of patients showed this discordance pattern in our study. This condition reflects persistent peripheral IGF-1 overproduction despite apparently adequate suppression of GH and could be explained by several mechanisms. First, GH pulsatility does not always correlate with IGF-1 levels and minor elevation in GH pulses can lead to elevated IGF-1 levels despite GH appears normal on nadir measurements. In addition, individual differences in peripheral IGF-1 synthesis may prolong IGF-1 elevation [3].

Elevated GH with normal IGF-1 levels is less common and may be due to reduced hepatic IGF-1 synthesis, as seen in conditions such as liver dysfunction, malnutrition, treatment with oestrogen, or poorly controlled diabetes. In addition, differences in reference ranges, assay variability, and timing of postoperative assessment may further contribute to the risk of biochemical discordance [3, 7].

In our study, no significant differences were observed between the discordant and the concordant group in baseline variables at diagnosis: age, gender, IGF-1, invasive tumour, micro/macro-adenoma or BMI. There were also no differences in antihypertensive treatment at time of surgery, nor in the rates of postoperative radiotherapy or reoperation. However, a significant difference in preoperative IGF-1 at diagnosis was identified between the discordant and the subgroup of patients with controlled concordant hormone profile. The proportion of macroadenomas and invasive tumours (Table 3) were: in the discordant group 87% and 47%, in the controlled group 69% and 23% and in the uncontrolled group 88% and 73% respectively. A recently published article on biochemical discordance by D. Emmanouilidis et al. [6], including 156 patients with acromegaly surgically treated between 1984 and 2017, shows similarities to our data concerning clinical traits. Their cohort also presents with a trend of a larger portion of macroadenomas in the discordant group and presence of invasive tumours.

The proportion of discordant cases in our study is similar to existing data [3–7] (20% versus 19–35%), although in the lower range. The most common pattern of biochemical discordance is characterised by elevated IGF-1 and normal GH [7]. A study investigating postoperative biochemical change without additional therapy in patients with acromegaly [23], reported that the switch of biochemical status occurred within the first year in two thirds of cases. If this time frame affects the risk of biochemical discrepancy being detected before a switch to concordance, it may have affected our rates. In the early part of the period in our study, some patients (six of them) first underwent a follow-up OGTT about one year post-surgery according to clinical practice at the time. The cut-off for GH used for defining a pathological value should also reasonably impact the frequency of biochemical discordance.

In this study, the majority of patients with discordant GH and IGF-1 were men (75%). Conversely, Brzana et al. [3] showed that 77% of patients in their group of high IGF-1 and normal GH pattern were women. It is well known that oestrogen replacement therapy via the oral route, elevates GH and lowers IGF-1 by oestrogen’s suppressive effect on the liver’s production of IGF-1 [13]. However, no patient in the discordant group were on oestrogen replacement therapy, either in the study by Brzana et al. [3] or in our study. Regardless, since our discordant cohort was predominantly male, this effectively rules out the ‘oestrogen effect’ as a driver in our population. Noteworthy is also the phenotypic stability that was observed in our cohort: only one patient switched discordance type over the study period. This suggests that post-surgical discordance is not a transient fluctuation or measurement error, but a stable biological state, potentially reflecting the intrinsic secretory characteristics or ‘set-point’ of the remnant tumour tissue or that other factors affect the IGF-1 levels.

In our study, the frequency of antihypertensive treatment did not differ between the groups. Similarly, a study by Romanisio et al. with 18 patients surgically treated for acromegaly with 7 years follow-up [24] presented no difference in prevalence of diabetes or hypertension between their discordant and concordant group. In another article [25] of 190 surgically treated patients with acromegaly, there was no difference between the discordant and the concordant group with regard to number of antihypertensives or treatment for diabetes mellitus. Concerning BMI, there was no difference in median BMI between our groups even though other studies have reported BMI as a risk factor for discordant values [15]. However, BMI is not an accurate measurement of body composition, as it does not distinguish between lean body mass and fat mass [26]. This aspect of the cohort was not investigated in detail, nor were potential ethnical differences within the cohort considered, which may have influenced the result.

The strength of this study is that the data consists of a large cohort of patients with acromegaly from the same region, surgically treated at a Pituitary Centre of Excellence with medical records of detailed clinical information. The retrospective design of this work presents with expected limitations such as: patients being examined with different GH and IGF-1 assays, varying consensus criteria for diagnosis and treatment evaluation in clinic during the study period, and patients were not evaluated with a standardised form for clinical assessment of acromegalic symptoms (which could have contributed to understanding if discordant hormones caused clinical symptoms).

Early postoperative assessment of GH and IGF-1 levels may have resulted in an overestimation of the frequency of biochemical discordance, as IGF-1 normalisation can take several months after surgery. The current recommendation is to evaluate biochemical control three months after surgery [27] but historically the guidelines have include recommendations of earlier evaluation, affecting the treatment of a few patients in the current study [28]. The specific aim of our study was to characterise the group of patients exhibiting biochemical discordance post-surgery within the set study period and to investigate potential clinical predictors of this pattern.

Overall, the relationship between hormone discordance and disease control remains unclear and therefore also its clinical implications. The reason could be related to limitations of the study such as: the patient cohort was too small to detect a difference in the examined variables or the examined variables do not drive the persistence of biochemical discordance between GH and IGF-1.

Given that prolonged overexposure to GH and IGF-1 [29, 30] increases morbidity and mortality in patients with acromegaly, it is crucial to further improve the understanding of discordant hormone profiles in acromegaly and its potential effects on patients’ outcomes. Our study highlights that about 1 in 5 patients in a cohort of surgically treated patients with acromegaly may exhibit biochemical discordance. Limited data exists on long-term effects of this hormonal state, making management and treatment decisions challenging in this patient group.

The current study cannot provide recommendations for optimal management of patients with acromegaly and biochemical discordance. Treatment decisions should be individualised, taking into account not only biochemical data, but also symptom burden, residual tumour, and risk of complications. Elevated IGF-1 levels in the context of significant disease burden may warrant early treatment, whereas a watchful waiting approach could be appropriate in selected cases. Further studies are however needed to evaluate the impact of biochemical discordance on long-term outcomes in acromegaly and to provide clinical guidance for treatment decisions.

## Conclusion

In this single-centre study, biochemical discordance after pituitary surgery occurred in one fifth of patients with acromegaly. The predominant pattern was an elevated IGF-1 with a normal GH nadir and most patients maintained the same discordance type. No significant differences in baseline clinical characteristics were identified between the discordant and controlled concordant group except for preoperative IGF-1 levels at diagnosis, which were significantly higher in patients with discordance. This suggests that higher preoperative disease activity (IGF-1 burden) may indicate a tumour biology that is less amenable to complete surgical remission. These patients effectively inhabit a grey zone between cure and active disease, warranting vigilant, individualised monitoring rather than a binary ‘cured/not cured’ classification. Trends towards macroadenomas and invasive tumours within the discordant group together with the limited data on effects of the hormone discordance, calls for a comprehensive postoperative follow-up involving a longitudinal assessment of clinical status, laboratory results and comorbidities to evaluate remission status.

## Data Availability

Upon reasonable request, data can be made available in consideration of research ethical aspects.

## References

[1] Fleseriu M, Langlois F, Lim DST, Varlamov EV, Melmed S (2022) Acromegaly: pathogenesis, diagnosis, and management. lancet Diabetes Endocrinol 10(11):804–826 36209758 10.1016/S2213-8587(22)00244-3

[2] Giustina A, Colao A (2025) Acromegaly. N Engl J Med 393(19):1926–1939 41223366 10.1056/NEJMra2409076

[3] Brzana JA, Yedinak CG, Delashaw JB, Gultelkin HS, Cook D, Fleseriu M (2012) Discordant growth hormone and IGF-1 levels post pituitary surgery in patients with acromegaly naïve to medical therapy and radiation: what to follow, GH or IGF-1 values? Pituitary 15(4):562–570 22183781 10.1007/s11102-011-0369-1

[4] Alexopoulou O, Bex M, Abs R, T’Sjoen G, Velkeniers B, Maiter D (2008) Divergence between growth hormone and insulin-like growth factor-i concentrations in the follow-up of acromegaly. J Clin Endocrinol Metab 93(4):1324–1330 18230660 10.1210/jc.2007-2104

[5] Bianchi A, Giustina A, Cimino V, Pola R, Angelini F, Pontecorvi A et al (2009) Influence of growth hormone receptor d3 and full-length isoforms on biochemical treatment outcomes in acromegaly. J Clin Endocrinol Metab 94(6):2015–2022 19336510 10.1210/jc.2008-1337

[6] Emmanouilidis D, Polanski W, Juratli T, Sobottka SB, Tsourdi E, Gruber M et al (2025) Predictive factors for post-therapeutic biochemical discordance in acromegaly: a monocentric analysis of 156 cases. Pituitary 28(4):74 40544415 10.1007/s11102-025-01547-8 PMC12183128

[7] Kanakis GA, Chrisoulidou A, Bargiota A, Efstathiadou ZA, Papanastasiou L, Theodoropoulou A et al (2016) The ongoing challenge of discrepant growth hormone and insulin-like growth factor I results in the evaluation of treated acromegalic patients: a systematic review and meta-analysis. Clin Endocrinol 85(5):681–688 10.1111/cen.13129 27292418

[8] Peixe C, Sánchez-García M, Grossman AB, Korbonits M, Marques P (2022) Biochemical discrepancies in the evaluation of the somatotroph axis: Elevated GH or IGF-1 levels do not always diagnose acromegaly. Growth Horm IGF Res 64:101467 35609487 10.1016/j.ghir.2022.101467

[9] Schilbach K, Strasburger CJ, Bidlingmaier M (2017) Biochemical investigations in diagnosis and follow up of acromegaly. Pituitary 20(1):33–45 28168377 10.1007/s11102-017-0792-z

[10] Arafat AM, Möhlig M, Weickert MO, Perschel FH, Purschwitz J, Spranger J et al (2008) Growth hormone response during oral glucose tolerance test: the impact of assay method on the estimation of reference values in patients with acromegaly and in healthy controls, and the role of gender, age, and body mass index. J Clin Endocrinol Metab 93(4):1254–1262 18171702 10.1210/jc.2007-2084

[11] Oh Y (2012) The insulin-like growth factor system in chronic kidney disease: pathophysiology and therapeutic opportunities. Kidney Res Clin Pract 31(1):26–37 26889406 10.1016/j.krcp.2011.12.005 PMC4715090

[12] Chen JW, Nielsen MF, Caumo A, Vilstrup H, Christiansen JS, Frystyk J (2006) Changes in bioactive IGF-I and IGF-binding protein-1 during an oral glucose tolerance test in patients with liver cirrhosis. Eur J Endocrinol 155(2):285–292 16868142 10.1530/eje.1.02218

[13] Shoung N, Ho KKY (2023) Managing estrogen therapy in the pituitary patient. J Endocr Soc 7(5):bvad051 37143694 10.1210/jendso/bvad051 PMC10153416

[14] Paisley AN, Hayden K, Ellis A, Anderson J, Wieringa G, Trainer PJ (2007) Pegvisomant interference in GH assays results in underestimation of GH levels. Eur J Endocrinol 156(3):315–319 17322491 10.1530/eje.1.02341

[15] Zhang S, Li Y, Guo X, Gao L, Lian W, Yao Y et al (2018) Body mass index and insulin-like growth factor 1 as risk factors for discordant growth hormone and insulin-like growth factor 1 levels following pituitary surgery in acromegaly. J Formos Med Association = Taiwan yi zhi 117(1):34–41 10.1016/j.jfma.2017.02.014 28341329

[16] Campana C, Cocchiara F, Corica G, Nista F, Arvigo M, Amarù J et al (2021) Discordant GH and IGF-1 results in treated acromegaly: impact of GH cutoffs and mean values assessment. J Clin Endocrinol Metab 106(3):789–801 33236108 10.1210/clinem/dgaa859

[17] Shin MS, Yu JH, Choi JH, Jung CH, Hwang JY, Cho YH et al (2013) Long-term changes in serum IGF-1 levels after successful surgical treatment of growth hormone-secreting pituitary adenoma. Neurosurgery 73(3):473–479 23728452 10.1227/01.neu.0000431480.87160.84

[18] Pennlund A, Esposito D, Bontell TO, Skoglund T, Hallén T, Carén H et al (2025) Long-term clinical outcome of 103 patients with acromegaly after pituitary surgery. Pituitary 28(2):33 39987353 10.1007/s11102-025-01503-6 PMC11846723

[19] Giustina A, Barkan A, Casanueva FF, Cavagnini F, Frohman L, Ho K et al (2000) Criteria for cure of acromegaly: a consensus statement. J Clin Endocrinol Metab 85(2):526–529 10690849 10.1210/jcem.85.2.6363

[20] Giustina A, Chanson P, Bronstein MD, Klibanski A, Lamberts S, Casanueva FF et al (2010) A consensus on criteria for cure of acromegaly. J Clin Endocrinol Metab 95(7):3141–3148 20410227 10.1210/jc.2009-2670

[21] Trainer PJ, Barth J, Sturgeon C, Wieringaon G (2006) Consensus statement on the standardisation of GH assays. Eur J Endocrinol 155(1):1–2 16793942 10.1530/eje.1.02186

[22] James PT, Leach R, Kalamara E, Shayeghi M (2001) The worldwide obesity epidemic. Obes Res 9(Suppl 4):228s–33s 11707546 10.1038/oby.2001.123

[23] Espinosa-de-Los-Monteros AL, Sosa E, Cheng S, Ochoa R, Sandoval C, Guinto G et al (2006) Biochemical evaluation of disease activity after pituitary surgery in acromegaly: a critical analysis of patients who spontaneously change disease status. Clin Endocrinol 64(3):245–249 10.1111/j.1365-2265.2006.02430.x 16487431

[24] Romanisio M, Pitino R, Ferrero A, Pizzolitto F, Costelli S, Antoniotti V et al (2023) Discordant biochemical parameters of acromegaly remission do not influence the prevalence or aggressiveness of metabolic comorbidities: a single-center study. Front Endocrinol 14:1256975 10.3389/fendo.2023.1256975 PMC10565344 37829686

[25] Amodru V, Petrossians P, Colao A, Delemer B, Maione L, Neggers S et al (2020) Discordant biological parameters of remission in acromegaly do not increase the risk of hypertension or diabetes: a study with the Liege Acromegaly Survey database. Endocrine 70(1):134–142 32562181 10.1007/s12020-020-02387-1

[26] Romero-Corral A, Somers VK, Sierra-Johnson J, Thomas RJ, Collazo-Clavell ML, Korinek J et al (2005) Accuracy of body mass index in diagnosing obesity in the adult general population. International journal of obesity 2008;32(6):959 – 66 10.1038/ijo.2008.11 PMC2877506 18283284

[27] Melmed S, di Filippo L, Fleseriu M, Mercado M, Karavitaki N, Gurnell M et al (2025) Consensus on acromegaly therapeutic outcomes: an update. Nat reviews Endocrinol 21(11):718–737 10.1038/s41574-025-01148-2 40804505

[28] Melmed S, Jackson I, Kleinberg D, Klibanski A (1998) Current treatment guidelines for acromegaly1. J Clin Endocrinol Metabolism 83(8):2646–2652 10.1210/jcem.83.8.4995 9709926

[29] Esposito D, Ragnarsson O, Johannsson G, Olsson DS (2020) Prolonged diagnostic delay in acromegaly is associated with increased morbidity and mortality. Eur J Endocrinol 182(6):523–531 32213651 10.1530/EJE-20-0019

[30] Giustina A, Fleseriu M (2026) Acromegaly complications: an update. The Journal of clinical endocrinology and metabolism 10.1210/clinem/dgag114 PMC13235294 42014041

